# Climate change-driven shifts in the global distribution of tomato and potato crops and their associated bacterial pathogens

**DOI:** 10.3389/fmicb.2025.1520104

**Published:** 2025-01-30

**Authors:** Muhammad Hubab, Niloufar Lorestani, Rogayah Akram Mheisin Al-Awabdeh, Farzin Shabani

**Affiliations:** College of Arts and Sciences, Qatar University, Doha, Qatar

**Keywords:** climate change, pathogen management, *Solanum lycopersicum*, *Solanum tuberosum*, *Clavibacter michiganensis*, *Ralstonia solanacearum*, crop pathogen interaction

## Abstract

**Introduction:**

Climate change is increasingly affecting the global distribution and productivity of critical food crops, including *Solanum lycopersicum* (tomato) and *Solanum tuberosum* (potato). In particular, bacterial pathogens such as *Clavibacter michiganensis* and *Ralstonia solanacearum* are expected to shift their geographic ranges, posing new risks to these crops. This study hypothesizes that under future climate scenarios, the geographic overlap between these crops and their pathogens will increase in certain regions, leading to heightened agricultural risks, especially in areas currently considered safe from these pathogens.

**Methods:**

To test our hypotheses, the objective was to evaluate the potential impact of climate change on the geographic distribution of two key food crops (tomato and potato) and their bacterial pathogens for the current time and by 2050. This study used four species distribution models (SDMs) to predict current and future habitat suitability for both tomato and potato crops, as well as their associated pathogens, under two shared socioeconomic pathways (SSP4.5 and SSP8.5) and four global circulation models (GCMs).

**Results:**

The models projected significant poleward shifts in suitable habitats for tomatoes and potatoes, with notable expansions in higher-latitude regions such as Canada, northern Europe, and Russia, and contractions in current major production zones such as the United States (US), Brazil, parts of Africa, and China. For *Clavibacter michiganensis*, the overlap with tomatoes was substantial, whereas the overlap between potatoes and *Ralstonia solanacearum* was comparatively smaller.

**Discussion:**

Our hypothesis was partially supported by the results. While the overall overlap between crop and pathogen habitats remains limited, the risk areas for both pathogens are expected to expand under future climate conditions in regions such as eastern Australia, Japan, Spain, and France. These findings underscore the importance of region-specific agricultural planning and pathogen management strategies to mitigate the risks posed by climate change. Future efforts should focus on vulnerable areas to prevent significant economic losses and ensure food security.

## 1 Introduction

Tomatoes (*Solanum lycopersicum*) and potatoes (*Solanum tuberosum*) are essential crops due to their widespread consumption and economic value. Potatoes are the third most important food crop globally, with an annual production of over 330 million tons (FAO, 2016). They play a crucial role in food security, particularly in developing regions, where they serve as a key source of income (Lutaladio and Castaldi, 2009; Lutz and Kc, 2010). Similarly, tomatoes contribute 16% to global vegetable production, with a market value of $58 billion, making them a vital crop for local consumption and export (FAO, 2016). Tomatoes are rich in essential nutrients such as vitamin C and potassium and contain antioxidants such as lycopene, which are associated with reduced cancer risks and heart disease (Blanca et al., 2015; Mercado-Meza et al., 2023). Major producers of these crops include China, India, and the US, where they are cultivated across various climatic conditions (Costa and Heuvelink, 2018).

*Ralstonia solanacearum* (hereafter *Ralstonia*) and *Clavibacter michiganensis* (*Clavibacter*) are two major bacterial pathogens that infect plants, particularly members of the Solanaceae family, including potatoes and tomatoes (Fernandez-Pozo et al., 2015). *Ralstonia*, the causal agent of bacterial wilt, infects over 200 plant species, including tomatoes and potatoes. The pathogen enters the plant through the roots, colonizing the xylem vessels and causing wilting, yellowing of the leaves, and necrosis (Salanoubat et al., 2002). It thrives in warm, humid climates, particularly in tropical and subtropical regions such as Sub-Saharan Africa, where it persists in soil, plant debris, and water, making it difficult to control (Hayward and Lederberg, 2000).

*Clavibacter*, which causes bacterial canker in tomatoes, infects plants through natural openings and wounds. Nevertheless, there are currently no highly effective control strategies against this pathogen, primarily due to its ability to enter plants through natural openings, causing cankers on leaves and stems, infecting the xylem, and leading to wilting and necrosis (Chalupowicz et al., 2017). First identified in North America in 1910, the pathogen has since spread to all major tomato-growing regions globally. Its spread had been particularly severe in Europe, significantly affecting tomato crop yields (Baysal et al., 2011). Both pathogens pose serious threats to crop production, resulting in significant economic losses globally, as they affect the quality and yield of tomatoes and potatoes.

Climate change poses significant challenges to global agriculture, particularly for crops such as tomatoes and potatoes. Rising global temperatures and altered precipitation patterns are major threats to agricultural productivity. Higher temperatures accelerate plant stress, reduce crop fertility, and increase pests and disease prevalence (Abbass et al., 2022; Farooq et al., 2022; Nyamukondiwa et al., 2022). Potatoes, for example, thrive within an optimal temperature range of 5°C to 30°C, and temperatures above this threshold reduce tuber formation and overall yield (Haverkort and Verhagen, 2008; Levy and Veilleux, 2007). Reduced water availability, a consequence of climate change, further exacerbates these crops’ challenges as they are particularly sensitive to soil moisture deficits (Daccache et al., 2012). Similarly, tomato plants are sensitive to high temperatures, leading to reduced fertility, flower drop, and diminished fruit set when daytime temperatures exceed 29°C and nighttime temperatures rise above 21°C (Berry and Uddin, 1988).

Increased rainfall and humidity in some regions may also favor the spread of bacterial pathogens such as *Ralstonia* and *Clavibacter*, increasing the risk to tomato and potato production (Bhanwar, 2022; Singh et al., 2023). Moreover, climate change can extend the geographic range of these pathogens, exposing new areas to infection, particularly in temperate regions where these diseases were previously rare (Singh et al., 2023). In the face of such a changing scenario, it is necessary to predict the geographic patterns of host and bacteria’s range shifts caused by climate change.

Species distribution models (SDMs), also known as ecological niche models (ENMs), are widely used tools for predicting the suitable range of species under both current and future environmental conditions (Franklin, 2010; Guisan et al., 2013; Zurell et al., 2020). These models explore factors influencing species distributions by identifying statistical relationships between species occurrence and both biotic and abiotic predictors (Guisan et al., 2013). Despite their assumptions and uncertainties (Elith et al., 2010; Shabani et al., 2016a; Zurell et al., 2020), SDMs are widely used to predict the distribution of diverse taxa, including insects (Ward, 2007), plants (Araújo and Luoto, 2007; Williams et al., 2009; Shabani et al., 2016b), bacterial species such as *Bacillus thuringiensis* (Ejaz, 2025), and animals (Ahmadi et al., 2024; Lorestani et al., 2022; Luo et al., 2015; Peterson et al., 2001; Shabani et al., 2019). While some studies have predicted the future distributions of host species such as potatoes and tomatoes, along with their associated bacteria, in specific countries (Simon et al., 2010), global-scale predictions for both hosts and pathogens simultaneously remain rare in the scientific literature.

This study aims to model the current and future global distributions of both comprehensively *Solanum lycopersicum* and *Solanum tuberosum* alongside their bacterial pathogens, *Clavibacter michiganensis* and *Ralstonia solanacearum*. By utilizing state-of-the-art SDMs and incorporating climate projections under two shared socioeconomic pathways (SSP4.5 and SSP8.5), this research provides critical insights into how climate change will alter the spatial overlap between these crops and pathogens, identifying regions at increased risk of infection. We utilized four species distribution models (SDMs), including the generalized linear model (GLM), generalized boosting model (GBM), random forest (RF), and maximum entropy (MaxEnt), combined in an ensemble approach. For future projections, we used four global circulation models (GCMs): ACCESS-CM2, CMCC-ESM2, HadGEM3-GC31-LL, and MIROC6, focusing on two shared socioeconomic pathway scenarios developed for the Coupled Model Intercomparison Project Phase 6 (CMIP6): SSP4.5 and SSP8.5. The findings of this study are crucial for informing targeted agricultural interventions, guiding disease management strategies, and ensuring food security in the face of a rapidly changing climate. Given the global importance of tomatoes and potatoes, the results have far-reaching implications for policymakers, farmers, and researchers seeking to mitigate the impacts of climate change on crop production. The publication of this study is essential as it not only fills a critical knowledge gap in the field of agricultural and environmental science but also provides actionable insights that can help safeguard global food systems.

## 2 Materials and methods

### 2.1 Species data

**Figure 1:**
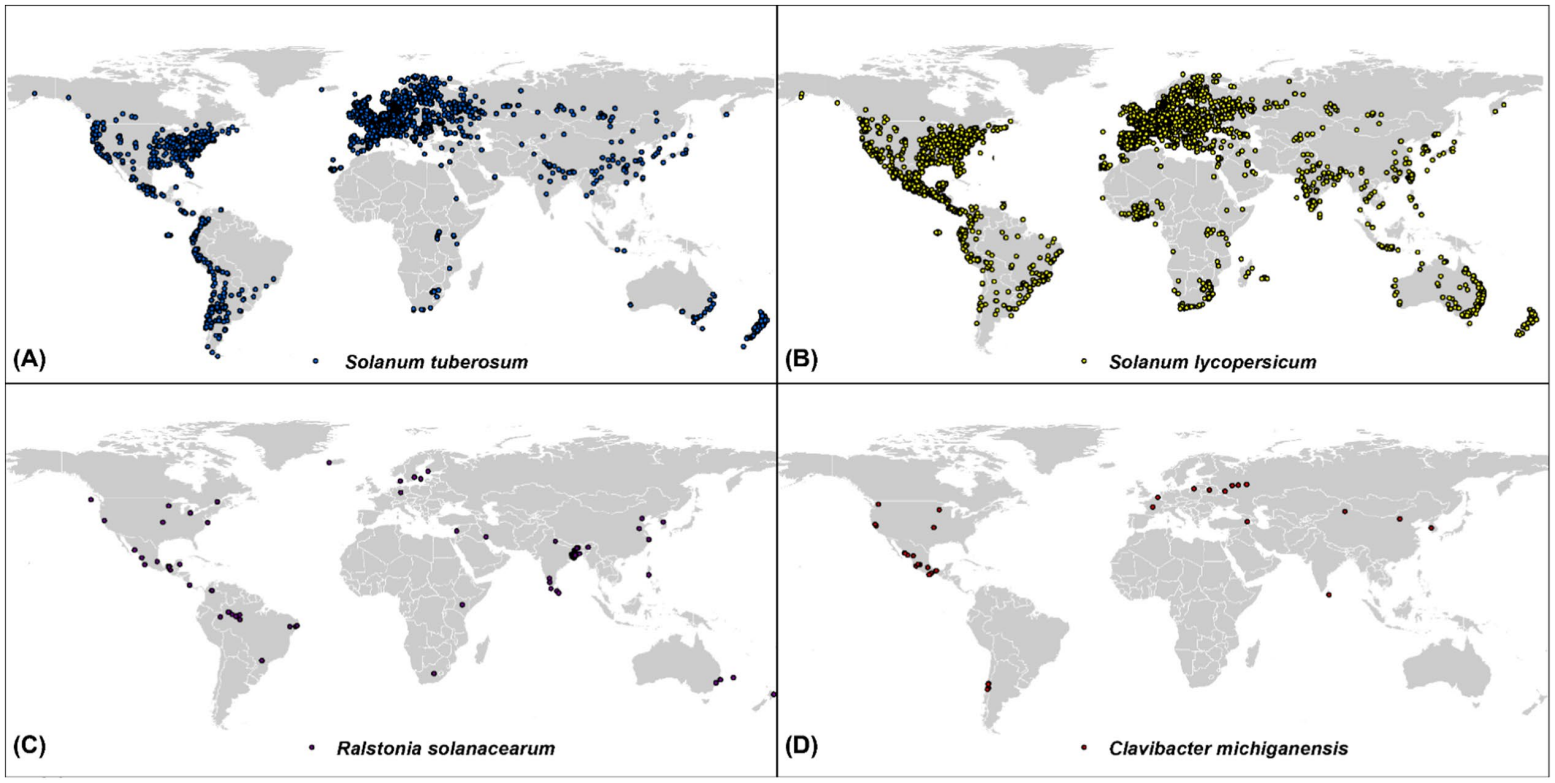
Global distribution of occurrence points for four species: *Solanum tuberosum* (potatoes, blue), *Solanum lycopersicum* (tomatoes, yellow), *Ralstonia solanacearum* (purple), and *Clavibacter michiganensis* (red). The occurrence data were compiled from the Global Biodiversity Information Facility (GBIF) and filtered for quality and accuracy.

Species data were collected from the Global Biodiversity Information Facility (GBIF) using the “rgbif” package within the R environment. GBIF is one of the largest open-source archives of biodiversity data in the world. However, before directly incorporating the GBIF data into SDMs, their precision, quality, and uncertainty should be assessed, as recommended by Zizka et al. (2019). To achieve this, we applied several filters to the GBIF data before downloading it. These filters removed duplicates, records without coordinates, potentially corrupt coordinates, data pre-dating 1990, coordinates with <4 decimals, and points with identical latitude and longitude (Zizka et al., 2019). In addition, for each species, we excluded occurrence points that were <5 km apart from each other, to reduce the adverse effects of spatial autocorrelation (SAC) (Dormann et al., 2007). Furthermore, data from greenhouse cultivation, protected environments, and controlled-temperature systems were excluded from the modeling process to ensure that only open-field occurrence points were retained. This resulted in 5,915; 7,946; 97; and 36 occurrence points for potatoes (*Solanum tuberosum*), tomatoes (*Solanum lycopersicum*), *Ralstonia solanacearum*, and *Clavibacter michiganensis*, respectively, which were used in the following analysis (Figure 1).

### 2.2 Environmental data

We obtained climate data for both current conditions and a future projection (2050) at a spatial resolution of 2.5 min (~4 Km2) from the WorldClim database (version 2.1). Given the potential multicollinearity among climatic variables, we employed the variance inflation factor (VIF) using the “usdm” package (Naimi, 2015). This analysis helped us identify the highly correlated climate variables (correlation exceeding 0.7). Following the VIF analysis, we selected eight factors as distribution predictors including bio1 (annual mean temperature), bio4 (temperature seasonality), bio5 (maximum temperature of the warmest month), bio6 (minimum temperature of the coldest month), bio12 (annual precipitation), bio13 = precipitation of the wettest month, bio14 (precipitation of the driest month), and bio15 (precipitation seasonality).

### 2.3 Modeling procedure

The potential current distributions of two hosts, namely, potatoes and tomatoes, and their associated bacteria were forecasted using an ensemble modeling technique (Araújo and New, 2007). We selected four SDMs including the generalized linear model (GLM), generalized boosting model (GBM), random forest (RF), and maximum entropy (MaxEnt) to develop the ensemble models, due to their widespread usage and high predictive performance compared to other methods (Phillips et al., 2006). All analyses were carried out using the BIOMOD2 package (Thuiller et al., 2009) in R Environment, version 4.3.2. The SDMs were performed separately for each host and bacteria, by using the same random sample of 10,000 background points. We employed a 10-fold cross-validation method for modeling. In this approach, presence points are randomly divided into 10 subsets. Then, models are constructed using 1 subsets for calibration and the remaining subsets for training, in each iteration. This process is repeated until all subsets have been used once for training. To measure the performance of individual models, we utilized the area under the curve (AUC) of the receiver operating characteristics (ROC) (Fielding and Bell, 1997), and the True Skill Statistic (TSS) (Allouche et al., 2006).

To assess the impact of climate change on the future distribution of four target species and compare their responses to climate change, climatic projections were conducted using four global circulation models (GCMs): ACCESS-CM2, CMCC-ESM2, HadGEM3-GC31-LL, and MIROC6. We focused on two shared socioeconomic pathway scenarios developed for the Coupled Model Intercomparison Project Phase 6 (CMIP6): SSP4.5 and SSP8.5. By employing multiple GCMs, we aimed to address the inherent uncertainties and limitations in climate modeling, providing a more comprehensive, and robust assessment of potential future climatic conditions suitable for the species. Similar to the current SDM, we integrated the model outputs into an ensemble model (Araújo and New, 2007). Through raster calculator analysis in ArcMap (v 10.80.2) for each SSP scenario, we averaged the obtained climatic suitability maps from the four GCMs. All these maps were converted to binary maps based on the threshold the maximum sum of sensitivity (Liu et al., 2016). The binary maps were overlaid to identify areas where hosts and bacteria could potentially coexist. By doing so, we generated the following scenarios:

- *Tomatoes and Potatoes at risk*: the distribution area of tomatoes and potatoes, where their ranges overlap with *Clavibacter michiganensis* and *Ralstonia solanacearum* bacteria.
- *Tomatoes and Potatoes without risk*: the distribution area of tomatoes and potatoes where *Clavibacter michiganensis* or *Ralstonia solanacearum* bacteria were absent.
- *Potatoes at risk*: the distribution area of potatoes, where their range overlaps with *Ralstonia solanacearum*.
- *Potatoes without risk*: the distribution area of potatoes, where *Ralstonia solanacearum* bacteria were absent.
- *Tomatoes at risk*: the distribution area of the tomatoes, where their range overlaps with *Clavibacter michiganensis*.
- *Tomatoes without risk*: the distribution area of tomatoes where *Clavibacter michiganensis* bacteria were absent.

In addition, to compare the differences in distributional changes of hosts and their bacteria, we calculated the three metrics for each: the number of grid cells that are suitable in both current and future time (stable), suitable in the current but will become unsuitable in the future (loss), and unsuitable in the current but will become suitable in the future (gain).

## 3 Results

Figure 1 shows the global distribution of occurrence for four species. Tomatoes (*Solanum lycopersicum*) are widely distributed across North and South America, Europe, parts of Asia, parts of Africa, Australia, and New Zealand, with dense concentrations in North America and central Europe. However, the distribution of *Clavibacter michiganensis*, which causes bacterial canker, is more geographically restricted, occurring mainly in temperate regions such as North and South America, Europe, and parts of Asia.

Potatoes (*Solanum tuberosum*) are distributed in North and South America, Europe, and across Asia, particularly in China. Meanwhile, *Ralstonia solanacearum* (the bacterial wilt pathogen) is concentrated in North and South America, Southeast Asia, and northern Europe. These regions where crop and pathogen distributions overlap are critical for understanding potential risk areas for infection.

### 3.1 Model performance

**Figure 2:**
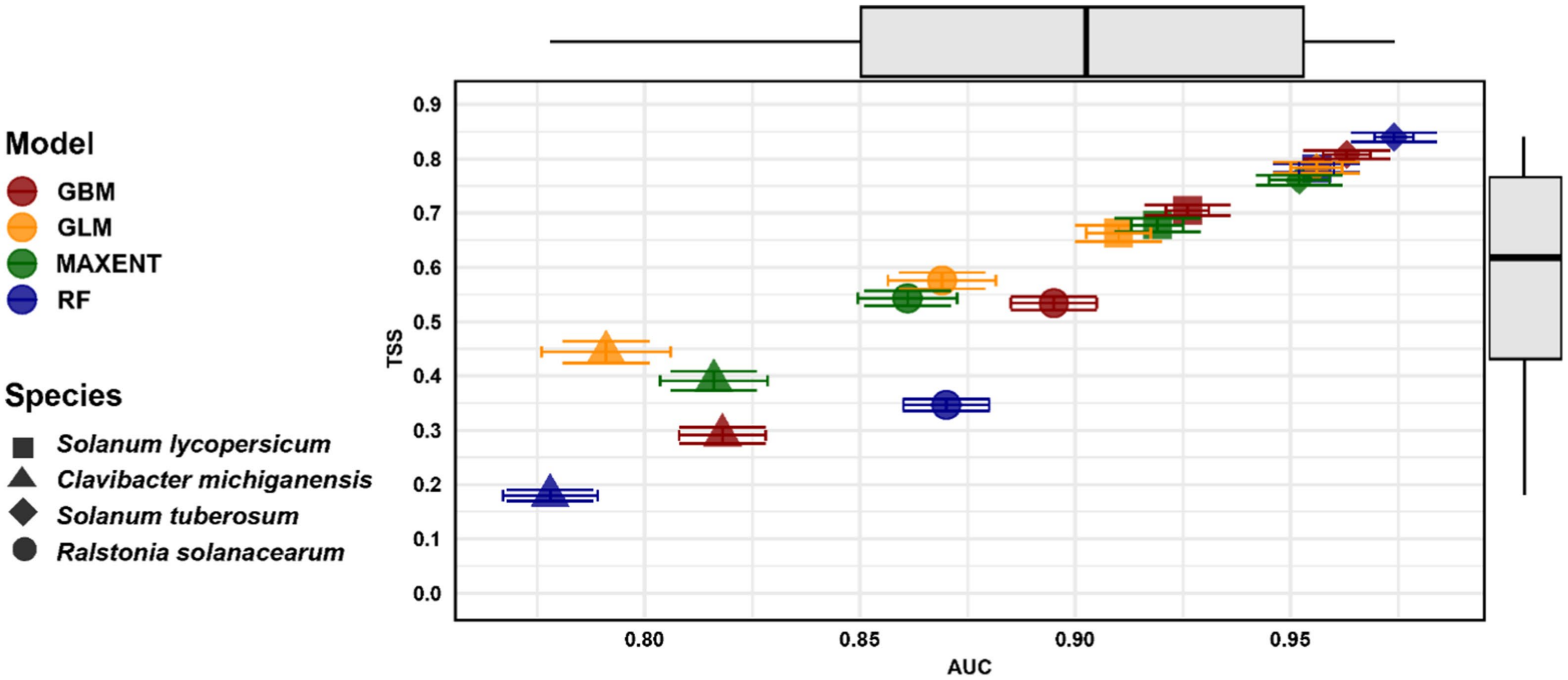
Mean AUC and TSS values for four species distribution models (GBM, GLM, MaxEnt, and RF) across four species: *Solanum lycopersicum* (tomatoes = Square), *Clavibacter michiganensis* (Triangle), *Solanum tuberosum* (potatoes = Diamond), and *Ralstonia solanacearum* (Circle). The error bars indicate the standard deviation of both AUC and TSS values (Refer to Supplementary Table S1 for detailed model performance data).

The SDMs for hosts and their associated bacteria showed a range of moderate-to-excellent predictive performances. Potatoes had the highest AUC and TSS scores of 0.974 and 0.84, respectively. In contrast, the lowest AUC and TSS values were observed for *Clavibacter michiganensis*, with AUC = 0.778 and TSS = 0.018. Comparing the predictive performance of four SDM methods across all species indicated that the RF model exhibited the highest performance (AUC = 0.974, TSS = 0.84), followed by GBM (AUC = 0.963, TSS = 0.808) and MaxEnt (AUC = 0.952, TSS = 0.760) (see Figure 2).

### 3.2 The comparative importance of climatic variables

For each species, we calculated the relative importance of climatic variables in their suitability model (see Table 1). For tomatoes and their pathogen *Clavibacter michiganensis*, the minimum temperature of the coldest month (bio6) had the highest contribution to climatic suitability, followed by the annual mean temperature (bio1). Similarly, for potatoes and their associated pathogen *Ralstonia solanacearum*, annual mean temperature (bio1) had the highest relative importance. The second most important variable for potatoes was the minimum temperature of the coldest month (bio6), while for *Ralstonia solanacearum*, the second most important variable was temperature seasonality (bio4).

**Table 1:** Mean and standard deviation (Sd) of the relative importance of climatic variables in the habitat suitability.

	Tomatoes		*Clavibacter michiganensis*		Potatoes		*Ralstonia solanacearum*	
	Mean	Sd	Mean	Sd	Mean	Sd	Mean	Sd
bio1	0.24	0.22	0.32	0.21	0.27	0.13	0.55	0.4
bio4	0.13	0.09	0.18	0.13	0.04	0.03	0.25	0.11
bio5	0.14	0.07	0.18	0.14	0.13	0.07	0.22	0.15
bio6	0.37	0.31	0.49	0.34	0.23	0.23	0.22	0.24
bio12	0.05	0.01	0.19	0.14	0.02	0.02	0.2	0.16
bio13	0.04	0.03	0.31	0.24	0.02	0.02	0.22	0.17
bio14	0.05	0.04	0.22	0.16	0.08	0.04	0.07	0.06
bio15	0.03	0.03	0.19	0.09	0.02	0.02	0.05	0.04

### 3.3 Current spatial distribution and habitat suitability for host crops and pathogens

**Figure 3:**
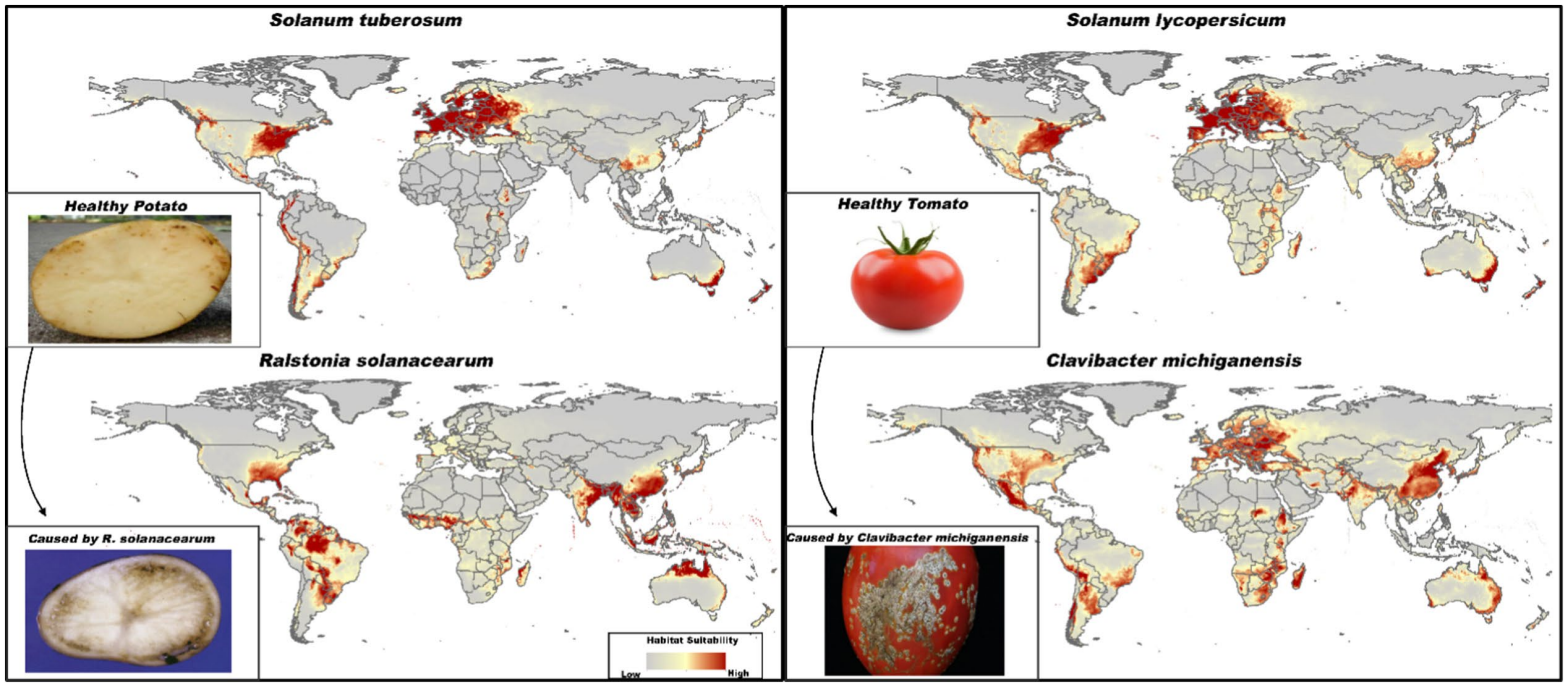
Predicted current suitable habitats for *Solanum tuberosum* (potatoes), *Solanum lycopersicum* (tomatoes), and their respective pathogens *Ralstonia solanacearum* and *Clavibacter michiganensis*. The color gradient represents habitat suitability, ranging from low suitability (gray) to high suitability (dark red). Images of tomato and potato were sourced from a Google search for illustrative purposes only (e.g., https://plantpath.ifas.ufl.edu/rsol/trainingmodules/BRPotato_PrinterFigures.html and https://pestadvisories.usu.edu/44-bacterial-canker-white-spots-tomato/).

Figure 3 illustrates the predicted suitable habitats for four species under current climatic conditions. The habitat suitability for tomatoes and their pathogen *Clavibacter michiganensis* shows significant overlap, particularly in most of Europe, South China, Eastern Australia, Southern Africa, and parts of North and South America. In contrast, the predicted suitable areas for potatoes and *Ralstonia solanacearum* differ somewhat. However, there is overlap in critical regions, including Southeastern America, Eastern Australia, Southeastern China, and parts of Africa.

### 3.4 Percentage gain and loss in the distribution of hosts and bacteria

Projecting the current SDMs to future climate change scenarios (2050 SSP4.5 and SSP8.5) reveals significant shifts in the suitable habitats for both host crops and their associated bacterial pathogens (refer to Supplementary Figures S1–S4 for continuous suitability maps of the four species separately).

**Figure 4:**
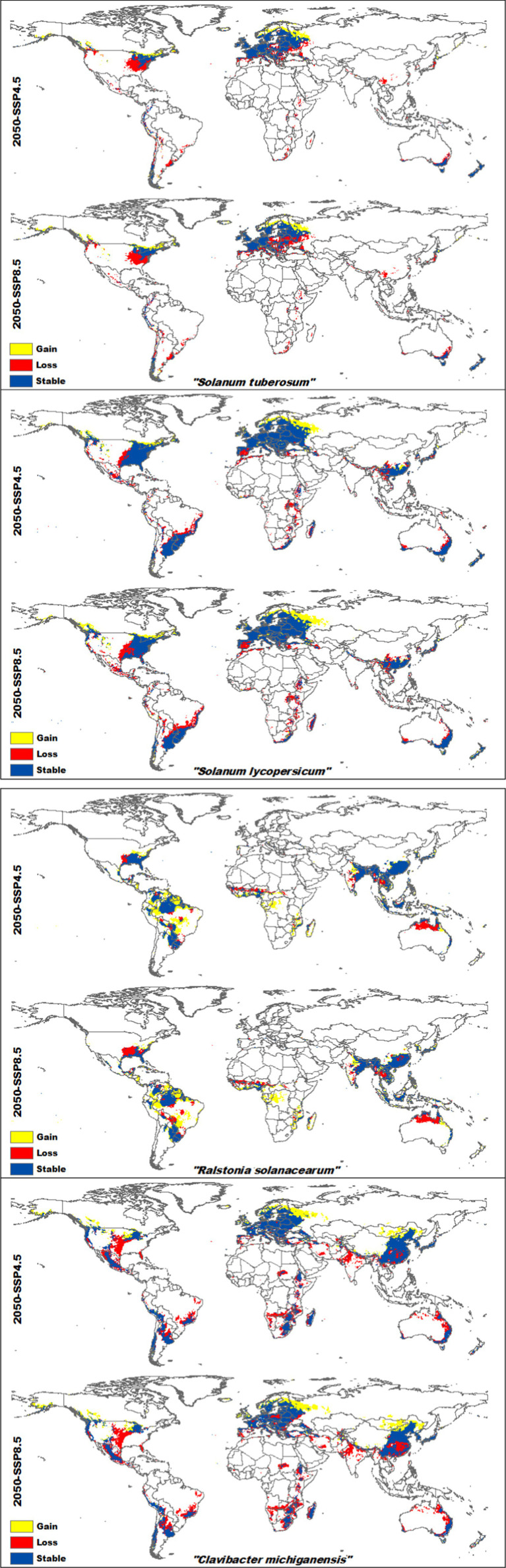
Projected future habitat changes (gain, loss, and stable areas) for *Solanum tuberosum* (potatoes), *Solanum lycopersicum* (Tomatoes), *Ralstonia solanacearum*, and *Clavibacter michiganensis* under climate change scenarios SSP4.5 and SSP8.5 by 2050. Yellow regions represent areas of habitat gain, red regions indicate habitat loss, and dark blue regions mark areas that remain suitable over time.

For potatoes, suitable habitats are expected to shift from western Europe to eastern Russia and from northwestern America toward Canada. However, potatoes will lose suitability in regions such as the US, Brazil, most countries in Africa, Spain, Portugal, Ukraine, Poland, China, Japan, and South Korea. In contrast, *Ralstonia solanacearum* bacteria will retain most of its current suitable areas in southern China, and parts of northern and northwestern Brazil, according to future scenarios. Although its future projections show expansion across all of Brazil and central Africa, there will be a loss of suitable areas in northern Australia and some parts of the US. Suitable areas for tomatoes are expected to expand from western Europe to western Russia and from northwestern America to Canada. However, significant habitat loss will occur in the US, Spain, most African countries, and parts of China and Australia. *Clavibacter michiganensis* follows a similar pattern, with wide coverage in Russia and Mongolia. While future projections indicate reductions in northern Australia, southeastern China, and parts of the Americas and Asia, there will still be substantial overlap between the areas suitable for both tomatoes and *Clavibacter michiganensis* (Figure 4). The highest habitat gain was observed in *Ralstonia solanacearum*, with an increase of 65.34% under SSP4.5 and 71.32% under SSP8.5 by 2050. It also experienced the lowest habitat loss, at 12.29% (SSP4.5) and 15.5% (SSP8.5). In contrast, tomatoes showed the least habitat gain, with 24.46% under SSP4.5 and 28.85% under SSP8.5. Potatoes had the highest habitat loss, with projected losses of 23.95% under SSP4.5 and 29.2% under SSP8.5 (Table 2).

**Table 2:** Projected percentage of habitat gain and loss for each species, under future climate scenarios SSP4.5 and SSP8.5 by 2050.

Species	Percentage loss		Percentage gain	
	SSP4.5	SSP8.5	SSP4.5	SSP8.5
Tomatoes	15.42	18.96	24.46	28.85
*Clavibacter michiganensis*	21.31	26.54	35.44	35.44
Potatoes	23.95	29.2	30.68	35.33
*Ralstonia solanacearum*	12.29	15.5	65.34	71.32

### 3.5 Overlay

**Figure 5:**
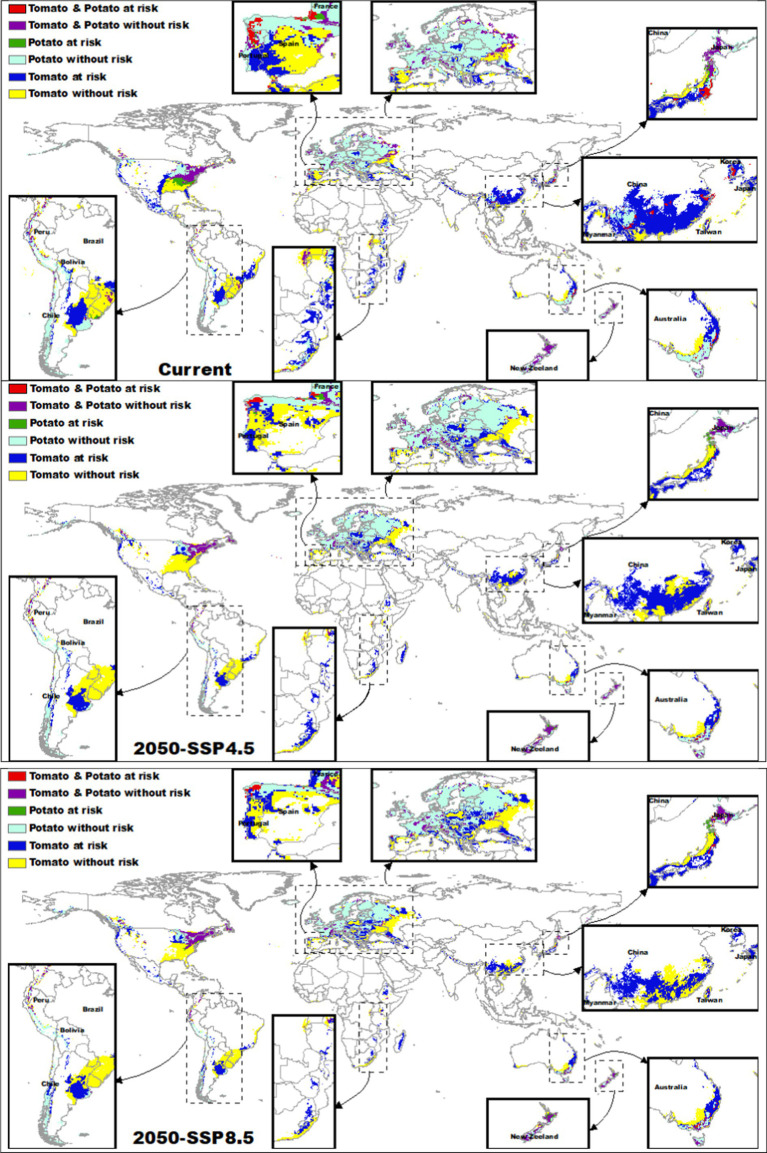
Projected global distribution of tomatoes and potatoes at risk from *Clavibacter michiganensis* and *Ralstonia solanacearum* under current and future climate scenarios (SSP4.5 and SSP8.5 for 2050). Red areas show the overlap of both pathogens with tomatoes and potatoes. Dark blue and green areas represent regions where only tomatoes or potatoes, respectively, are at risk. Yellow and light blue areas indicate regions where tomatoes and potatoes remain suitable without pathogen risk, while purple areas show regions without risk for either crop.

The red areas in Figure 5 represent regions where both tomatoes and potatoes are at risk due to overlapping suitable habitats with both *Clavibacter michiganensis* and *Ralstonia solanacearum*. Currently, these regions are concentrated in a few specific areas globally, including parts of eastern Australia, China, Japan, France, Spain, and Portugal. Collectively, these regions represent a relatively small fraction (~1%) of the total suitable habitat for both crops. However, future climate projections indicated a decline in the extent of these risk areas (SSP4.5 = 0.05%; SSP8.5 = 0.04%), particularly in China and Japan.

For tomato, areas at risk from *Clavibacter michiganensis* (represented in dark blue in Figure 5) cover a significantly larger extent than other risk areas. Currently, the overlap between tomatoes and *Clavibacter michiganensis* suitable habitats is approximately 61.11%. By 2050, this overlap is projected to decrease to 58.2% under SSP4.5 and 56.35% under SSP8.5, particularly in regions such as the US, Brazil, and Spain. Despite this projected reduction, significant risk areas will remain in southeastern Australia, Japan, China, eastern Africa, and Argentina, especially under the SSP8.5 scenario. Conversely, parts of Europe, the northeastern US, and southern Brazil are expected to remain without risk (depicted in yellow in Figure 5).

In contrast, potatoes show the lowest overlap with *Ralstonia solanacearum* (represented in green in Figure 5). Currently, the overlap between potatoes and this pathogen is only 5.36%, concentrated in regions such as the eastern US and parts of France. This overlap is projected to decrease further to 1.87% under SSP4.5, with residual risk areas remaining in France, and to 1.97% under SSP8.5, although some increase is projected in parts of Japan. Areas not at risk for potatoes (depicted in light blue in Figure 5) are projected to remain stable across most of Europe and the northeastern US, although decreases in suitability are expected in Australia and China.

Regions without risk from either *Clavibacter michiganensis* or *Ralstonia solanacearum* (represented in purple in Figure 5) are currently concentrated in eastern North America, New Zealand, central Japan, Russia, and parts of Europe, particularly France. These regions are expected to remain relatively stable in the future, particularly in the US, France, New Zealand, and Japan.

## 4 Discussion

This study assessed climate change impacts on the habitats of two key crops, tomatoes and potatoes, and their associated pathogens, *Clavibacter michiganensis* and *Ralstonia solanacearum*. The key focus of this analysis was to identify the most critical areas at risk for both crops using spatially explicit species distribution models (SDMs) under future climate change scenarios (SSP4.5 and SSP8.5). All SDMs demonstrated strong predictive performance across species. Random forest (RF) models demonstrated the highest accuracy for predicting potato distribution, providing robust support for our climate-based projections.

Temperature emerged as the dominant factor affecting potato distribution, corroborating previously mentioned findings on the crop’s sensitivity to temperature fluctuations (Champooiseau et al., 2008; Kim and Lee, 2019; Obiero et al., 2022; Rana et al., 2020). Increased temperatures negatively affect the growth, yield, and quality of potatoes. Key physiological processes such as net photosynthesis, tuber emergence, and leaf appearance rate are all impacted by temperature fluctuations. Studies suggest that while temperatures below 20°C delay sprout growth, temperatures above the optimal range also hinder sprout development in potatoes (refer to Singh et al., 2013 for more detail). However, these projections are based solely on climatic factors and do not account for critical agricultural variables such as soil type and land management practices, which may significantly affect real-world outcomes.

For tomatoes, the minimum temperature of the coldest month was the most important factor, consistent with previous research showing that extreme temperatures reduce tomato yield and fruit quality (Silva et al., 2017b), which has shown that high temperatures can lead to considerable yield losses due to reduced fruit set, smaller fruit size, and diminished fruit quality in tomatoes (Singh et al., 2013). Overall, climate change impacts, such as flooding and drought conditions, significantly affect tomato growth as this crop is highly sensitive to climate stress (Shabani et al., 2012; Silva et al., 2017a, 2017b). For example, water deficits can reduce fruit number and weight, induce flower abscission, and decrease reproductive activity (Heuvelink, 2005). Previous studies also highlighted the importance of other climatic variables; for example, annual precipitation (bio12) and precipitation of the driest quarter (bio17) have been identified as key environmental variables influencing the suitability of tomatoes in China (Liu et al., 2024). Suitable habitats for tomatoes are projected to shift northward (Silva et al., 2017a), with expansions in northern Europe and Russia, and contractions in traditional growing regions such as the US and Spain (Carrasco et al., 2021). The projected losses in Spain may be attributed to a decrease in the length of the crop season (LCS) for tomatoes, which is expected to shrink to 48 days by 2050, compared to a range of 76 to 180 days in 2000, as predicted by Saadi et al. (2014). Given these projected shifts, it is crucial for countries facing declining suitability for tomato cultivation, such as Mexico—considered the center of tomato domestication in America (Ramírez-Ojeda et al., 2022)—to incorporate these findings into agricultural planning.

Similarly, *Clavibacter michiganensis*, the pathogen that leads to bacterial canker, is projected to follow a similar spatial shift to regions such as western Russia, northeastern China, and central Mongolia. Some areas in Europe, southern Africa, and most of China are expected to remain suitable for bacterial canker under future climate scenarios. Based on our results and previous studies on tomato cultivation in these stable regions, such as the study by Liu et al. (2024) in China, a significant overlap between the future suitable habitats of tomatoes and their pathogen is likely to persist over time.

Our results indicated an ongoing shift in the suitable range for potatoes toward higher latitudes, from western Europe and North America to eastern Russia and Canada. We found that potato suitability decreases in regions such as the US, Brazil, several European countries, Africa, China, Japan, and South Korea, reflecting a global reduction in suitable habitats for this crop due to rising temperatures. However, Kim and Lee (2019) predicted that potato yields in South Korea, particularly for spring cultivation, are expected to increase under climate change. Nonetheless, they indicated that yield decreases in hotter regions and during high-temperature episodes remain a concern. Our findings also showed that southeastern Australia will remain suitable for potato cultivation over time. At the same time, *Ralstonia solanacearum* is expected to lose the majority of its suitable areas in Australia. However, potatoes in Australia remain at risk from other pests and pathogens, such as the tomato potato psyllid (TPP) and its associated bacterial plant pathogen *Candidatus Liberibacter solanacearum* (CLso), as reported in previous studies (Wan et al., 2020). In addition, *Ralstonia solanacearum* was projected to maintain its current range in southern China and parts of northern and southern Brazil, while expanding significantly across Brazil, Central Africa, and India.

Finally, after overlapping the suitable habitats of both host crops and their bacterial pathogens, we identified critical areas that may represent significant risks under both current and future climate scenarios. Although the total overlap regions constitute a relatively small fraction of the global habitat for both crops, agriculturally important regions such as eastern Australia, parts of China, Japan, Spain, Portugal, and France are projected to remain at risk for both pathogens, *Clavibacter michiganensis* and *Ralstonia solanacearum*, in both current and future scenarios. The greater overlap between tomatoes and *Clavibacter michiganensis*, compared to potatoes and *Ralstonia solanacearum*, suggests that tomato cultivation may face more substantial pathogen risks in the future, particularly in regions such as southeastern Australia, eastern Africa, Argentina, and much of China. Conversely, while potatoes show less overlap with *Ralstonia solanacearum*, notably in the US and Japan, other factors may still pose significant threats to potato cultivation. These results underscore the urgency of developing targeted management strategies in regions where crop–pathogen overlap is projected to increase, particularly for maintaining food security in vulnerable agricultural zones.

This study offers critical insights into the future distribution of tomatoes and potatoes under climate change, providing a foundation for informed agricultural planning. However, our models are based solely on climatic variables, without considering essential factors such as soil properties, land use, or pathogen–host competition. As a result, while our projections highlight potential broad-scale geographic shifts, real-world outcomes could vary significantly based on local environmental conditions and management practices. In addition, other pests and pathogens, such as the potato tuber moth (*Phthorimaea operculella*) (Jung et al., 2020), *Phytophthora infestans* (González-Jiménez et al., 2023), and *Neoleucinodes elegantalis* (Silva et al., 2017b), could further influence the suitability of these crops but were not included in this analysis. Future research should integrate non-climatic variables, biotic interactions, and other pests and pathogens to comprehensively assess crops’ suitability under changing climate conditions.

While our findings provide a valuable forecast of climate-driven habitat shifts for tomatoes and potatoes, it is important to note that our models are based solely on climatic factors. Other crucial variables, such as soil type, land use, and biotic interactions, which also impact crop and pathogen distributions, were not included. Therefore, the results should be interpreted as indicative of broad-scale geographic shifts, rather than precise forecasts at a local or regional level. Furthermore, the inherent uncertainties in future greenhouse gas emission levels, as represented by the two shared socioeconomic pathways (SSP4.5 and SSP8.5), introduce an additional layer of uncertainty to the predictions. The maps and results should thus be viewed with caution, recognizing that they are scenarios based on assumptions about future climate trajectories, and real-world outcomes could differ depending on how these variables evolve.

## 5 Conclusion

This study highlights the potential impacts of climate change on the distributions of *Solanum lycopersicum (tomatoes), Solanum tuberosum (potatoes)*, and their bacterial pathogens, *Clavibacter michiganensis and Ralstonia solanacearum*. By 2050, we identified critical areas where both crops and pathogens may experience significant shifts in habitat suitability, with expansions projected toward higher latitudes in regions such as northern Europe, Canada, and Russia and significant declines anticipated in traditional production zones such as parts of the US, Brazil, and China. These findings underscore the urgent need for targeted agricultural planning and disease management strategies to mitigate the risks posed by these shifts.

While our models provide a critical baseline for understanding broad-scale climatic suitability, they are limited by their reliance on climatic variables alone. Factors such as land use, soil characteristics, biotic interactions, and agricultural management practices, which significantly influence crop and pathogen distributions, were not included. These omissions highlight the need for future studies to integrate additional environmental, biological, and socioeconomic data to enhance model accuracy and applicability.

Despite these limitations, this research offers valuable insights for policymakers and agricultural stakeholders, serving as a foundation for proactive strategies to safeguard food security under changing climatic conditions.

## Data Availability

The original contributions presented in the study are included in the article/Supplementary material, further inquiries can be directed to the corresponding author/s.
