# Supplementary material for: Climate change-driven shifts in the global distribution of tomato and potato crops and their associated bacterial pathogens
Source: Front Microbiol. 2025 Jan 30;16:1520104. doi: 10.3389/fmicb.2025.1520104 (PMC11821613; doi:10.3389/fmicb.2025.1520104)
Supplement: Supplementary file 1 [file Data_sheet_1.docx]

**Tale S1.** Mean AUC and TSS values for four species (*Solanum lycopersicum*, *Clavibacter michiganensis*, *Solanum tuberosum*, and *Ralstonia solanacearum*) across four species distribution models (GBM, GLM, MAXENT, RF).

|  | **Model** | **GBM** | **GLM** | **MAXENT** | **RF** |
| --- | --- | --- | --- | --- | --- |
| *Solanum lycopersicum* | AUC_mean | 0.926 | 0.91 | 0.919 | 0.956 |
|  | TSS_mean | 0.705 | 0.663 | 0.678 | 0.783 |
| *Clavibacter michiganensis* | AUC_mean | 0.818 | 0.791 | 0.816 | 0.778 |
|  | TSS_mean | 0.291 | 0.444 | 0.391 | 0.018 |
| *Solanum tuberosum* | AUC_mean | 0.963 | 0.956 | 0.952 | 0.974 |
|  | TSS_mean | 0.808 | 0.784 | 0.761 | 0.84 |
| *Ralstonia solanacearum* | AUC_mean | 0.895 | 0.869 | 0.861 | 0.87 |
|  | TSS_mean | 0.534 | 0.576 | 0.543 | 0.347 |


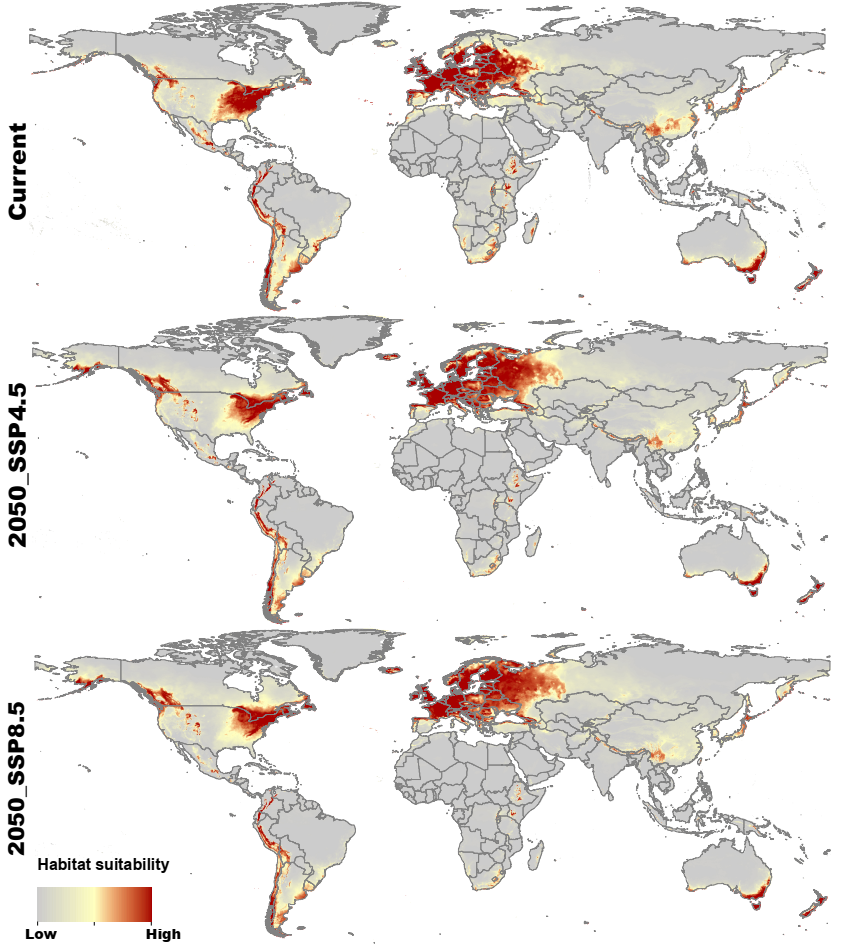


**Figure S1.** Continuous habitat suitability maps for Solanum tuberosum (potato) under current and future climate scenarios (SSP4.5 and SSP8.5) by 2050. The color gradient represents habitat suitability, ranging from low suitability (gray) to high suitability (dark red).


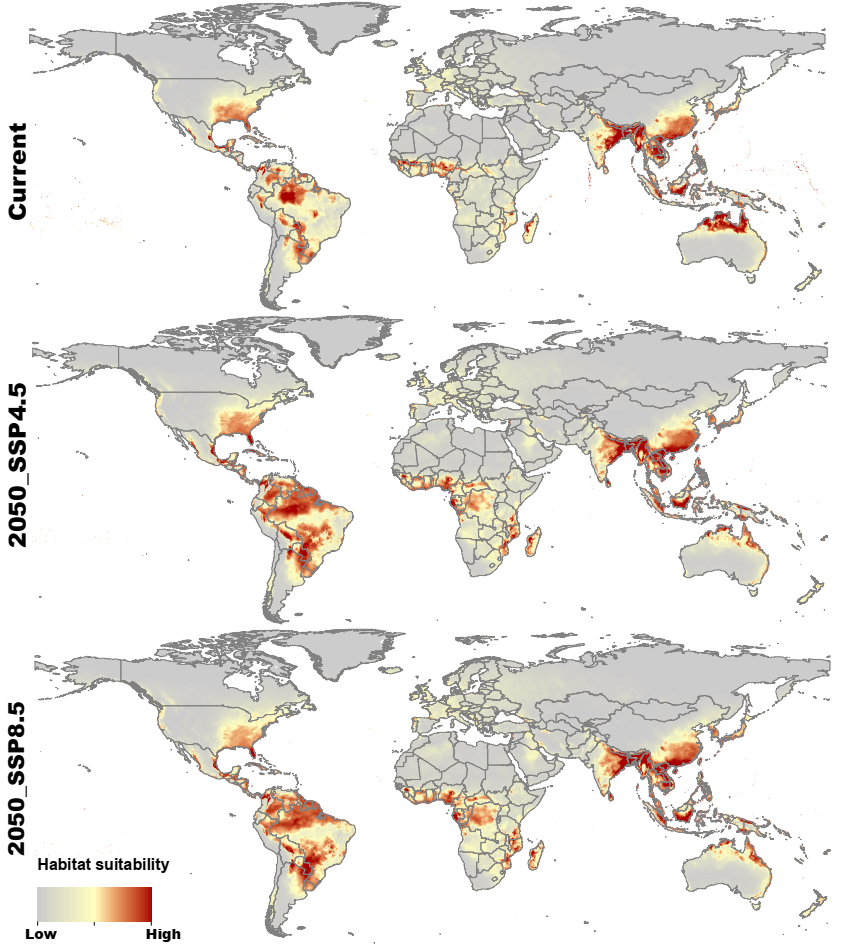


**Figure S2.** Continuous habitat suitability maps for Ralstonia solanacearum under current and future climate scenarios (SSP4.5 and SSP8.5) by 2050. The color gradient represents habitat suitability, ranging from low suitability (gray) to high suitability (dark red).


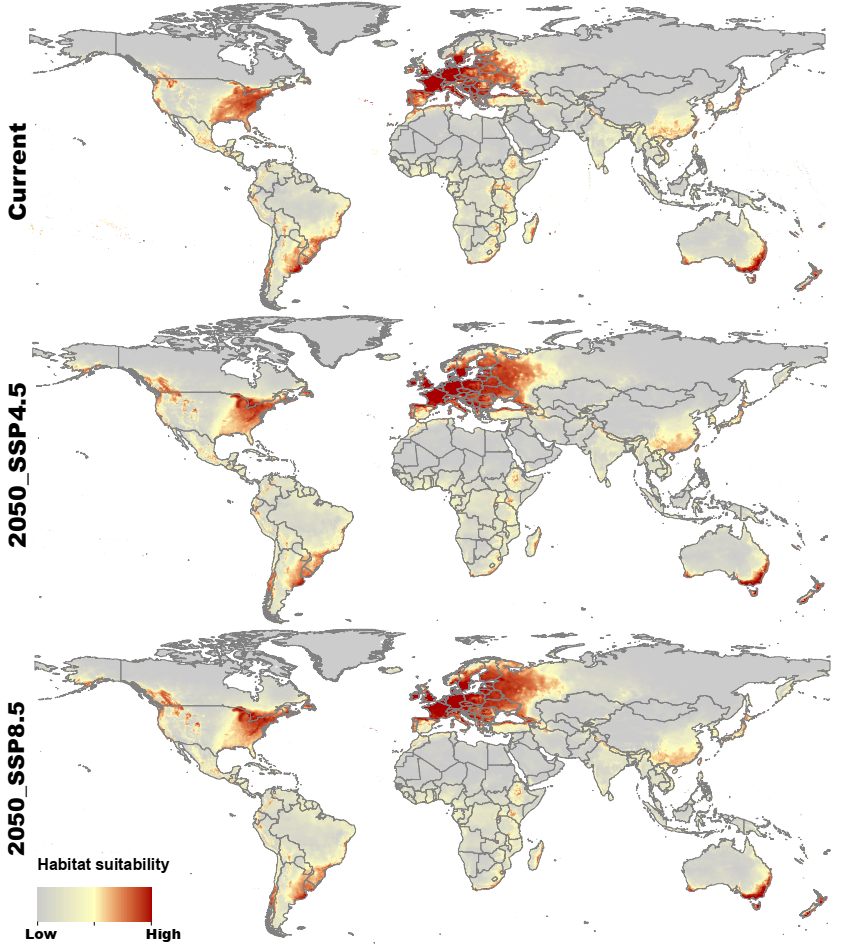


**Figure S3.** Continuous habitat suitability maps for Solanum lycopersicum (Tomato) under current and future climate scenarios (SSP4.5 and SSP8.5) by 2050. The color gradient represents habitat suitability, ranging from low suitability (gray) to high suitability (dark red).


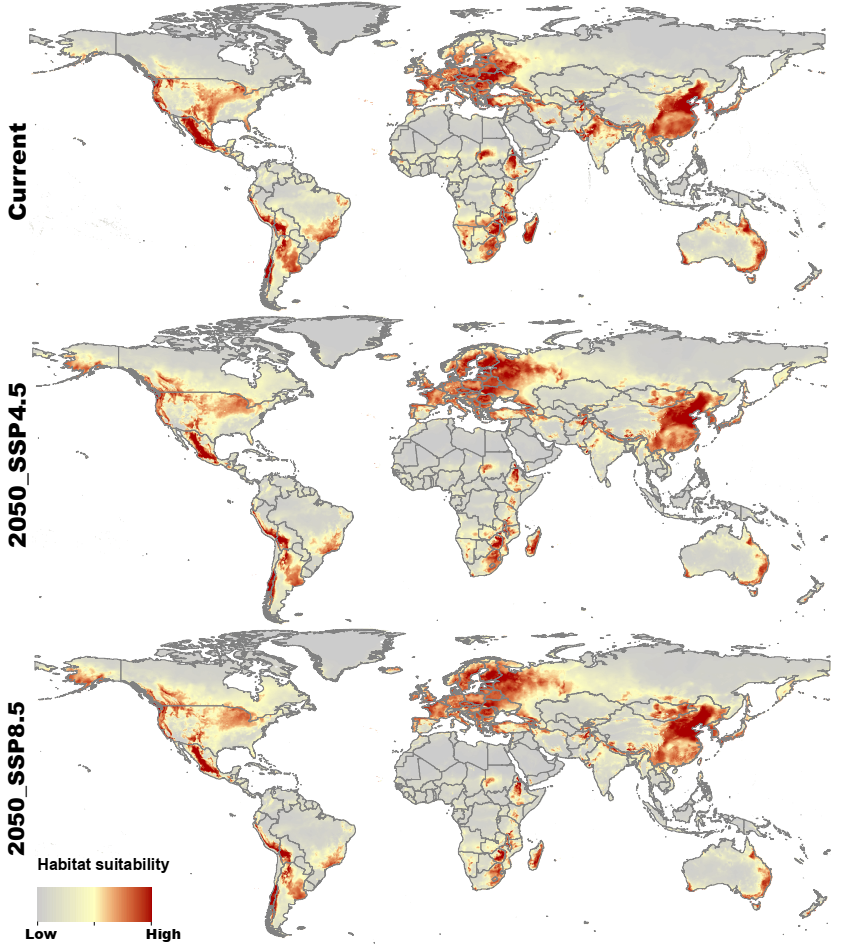


**Figure S4.** Continuous habitat suitability maps for Clavibacter michiganensis under current and future climate scenarios (SSP4.5 and SSP8.5) by 2050. The color gradient represents habitat suitability, ranging from low suitability (gray) to high suitability (dark red).
